# Assessing digital financial inclusion and financial crises: The role of financial development in shielding against shocks

**DOI:** 10.1016/j.heliyon.2024.e41231

**Published:** 2024-12-19

**Authors:** Huy Nguyen Quoc, Dinh Le Quoc, Hai Nguyen Van

**Affiliations:** Faculty of Finance and Accounting, Lac Hong University, Bien Hoa City, Dong Nai Province, Viet Nam

**Keywords:** Digital financial inclusion, Financial crisis, Financial stability, Financial development

## Abstract

Digital financial inclusion (DFI) has been proven to be a central factor in driving economic development and reducing inequality in countries. However, its impact on financial crises (FC) has yet to be clearly examined, particularly in the context of current Financial Development (FD). Therefore, this study examines the influence of DFI on FC across 52 countries from 2004 to 2020, focusing on how this impact varies with the level of FD. Using a combination of Threshold Regression (PTR) and Bayesian regression methods, the research first identifies structural breaks in the DFI-FC relationship, with FD as the threshold variable. Bayesian regression is then employed to address challenges such as small sample sizes, endogeneity, and autocorrelation, while assessing DFI's differential impact across countries with varying FD levels. The PTR analysis reveals a threshold value of 0.6036, indicating a non-linear DFI-FC relationship depending on FD levels. In low FD countries, DFI reduces the risk of FC, whereas in high FD countries, uncontrolled DFI increases it. Based on the results, we suggest that financial institutions should strengthen efforts to promote investment in digital technology by implementing digital skills training programs, providing capital to businesses and individuals, and facilitating support from financial institutions in countries with low FD. Meanwhile, for countries with high FD, regulatory authorities should establish stricter regulations and supervision mechanisms for digital financial activities to mitigate the risks of FC.

## 1 Introduction

This study evaluates the impact of Digital Financial Inclusion (DFI) within the context of financial crises (FC), focusing on the critical role of the threshold variable, Financial Development (FD). DFI refers to efforts to provide affordable financial services to underserved individuals and businesses through digital technologies such as mobile phones, the Internet, and electronic payment systems [1,2]. While DFI is a central element of development policy, historical events have linked it to FC. In Brazil, DFI programs have been extensively implemented through mobile payments. However, the increased use of technology has highlighted social disparities. Those lacking access to technology or financial literacy have been left behind, leading to increased inequality and exacerbating socio-economic issues. In Venezuela, the implementation of digital payment systems has faced numerous challenges related to cybersecurity and technical disruptions. Dependence on DFI has heightened the risk of asset loss and reduced confidence in the formal financial system, resulting in economic decline. A notable example of uncontrolled credit expansion is Sri Lanka in 2022. Prior to the COVID-19 pandemic, Sri Lanka already had high levels of debt. The shocks from the pandemic and the Russia-Ukraine war worsened the situation, leading to political, economic, and social crises. Sri Lanka struggled to collect foreign currency for importing essential goods, causing banking chaos and making it impossible for the country to continue borrowing due to reaching its debt ceiling. Over 50 % of households in Sri Lanka are in debt, leading to the country's sovereign default. In India, the DFI strategy adopts a comprehensive approach known as “extreme financial inclusion” [3]. Given the high population density and religious diversity, DFI provides access to formal financial services for everyone, including convicted individuals and criminals. Consequently, DFI in India is viewed as a source of financial instability. These observations raise concerns among researchers that the growing number of institutions and policies promoting DFI may pose risks that could lead to FC in some countries [4,5].

The expanding literature explores the relationship between Financial Inclusion (FI) and financial stability, with financial stability defined as the state in which the financial system—encompassing financial institutions, markets, and infrastructure—functions efficiently, allocates resources effectively, and remains resilient to various shocks [6,7].

However, the literature has yet to clearly analyze the relationship between DFI and FC, particularly the role of FD within this relationship. Specifically, Han and Melecky (2013) [8] argue that FI is related to financial stability because it provides a more diversified funding base for financial institutions through increased deposits from various sources, enhancing their resilience to shocks. Morgan and Pontines (2014) [9] highlight that FI leads to more effective savings intermediation and reduces the size of the informal economy, which benefits the stability of the financial system. García and José (2016) [10] also point out that FI facilitates the monitoring and enforcement of anti-money laundering and anti-terrorism financing laws, which are crucial for maintaining the integrity and stability of the financial system. Recently, Ozili (2024) [5] examined the impact of FI during FC and concluded that FI affects FC through the expansion of bank accounts, the number of ATMs, and the number of bank branches.

Understanding the impact of DFI on FC and examining the role of FD is crucial. This insight can help policymakers determine whether higher or lower levels of DFI contribute to a safer financial system or, conversely, increase vulnerability leading to FC. Additionally, assessing the impact of DFI can reveal whether excessive development of DFI is beneficial or poses risks, particularly concerning its effects on FC. If evidence shows that high levels of DFI lead to FC, it implies that too much of a good thing (in this case, DFI) may be detrimental. Such insights can guide policymakers in reassessing their national DFI strategies, focusing efforts on achieving an ‘optimal' level of DFI rather than pursuing a 100 % DFI goal that could destabilize the financial system. Based on the aforementioned arguments, the objective of this study is to evaluate the relationship between DFI and FC while considering the role of FD. To achieve the above objective, we collected data from countries around the world, addressing issues of data limitations. The remaining sample includes 52 countries, with the study period spanning from 2004 to 2020. The study employs two combined econometric methods. First, we use Threshold Regression (PTR), initially proposed by Hansen (1999) [11] and Wang (2015) [12], to describe abrupt changes or structural breaks in the relationship between variables. Based on the FD threshold, countries are divided into two groups: high financial development countries (HFDCs) and low financial development countries (LFDCs). Subsequently, we apply Bayesian Regression, which is advantageous for addressing issues related to small sample sizes, endogeneity, and autocorrelation [6,13], to examine the impact of DFI on FC within these classified country groups. The results indicate that in LFDCs, DFI reduces financial crisis risk, while in HFDCs, uncontrolled DFI increases risk. Based on these results, we recommend enhancing DFI in LFDCs and closely regulating DFI sources in HFDCs.

This paper contributes to the literature in three main ways. First, it provides both theoretical foundations and empirical evidence on the impact of DFI on FC, with FD considered as the threshold variable. Second, based on the analysis of the FD threshold, we categorize countries into groups to assess the impact of DFI on FC and offer relevant policy recommendations. Third, to address issues such as small sample sizes, autocorrelation, and endogeneity, we employ Bayesian Regression. Finally, based on the empirical results, we recommend that policymakers reassess their national DFI strategies and focus on identifying the ‘optimal' level of DFI.

The remainder of this article is organized as follows. The section "Literature Review" provides an overview of studies examining the relationship between DFI and FC. Following that, the section "Research Methodology" offers a brief introduction to the data, variable descriptions, rationale, and an overview of descriptive statistics. In the subsequent section, "Empirical Results," we delve into the findings of our research. Finally, the section "Conclusion and Policy Implications" concludes the article and presents tailored policy recommendations.

## 2 Literature review

### 2.1 Theoretical literature

The Diamond and Dybvig (1983) [14] theory on bank runs offers a valuable perspective for understanding the connection between DFI and FC, particularly in relation to economic cycles. The theory suggests that banks enter into agreements to provide services to both borrowers and depositors, with the latter having the right to access their funds at any moment. It also posits that a panic in the banking sector can spark a bank run if depositors become worried about the security of their funds [14]. During periods of economic downturn or financial instability, the likelihood of such panic increases, as economic uncertainty heightens depositors' fears. A bank run occurs when a large number of digitally included depositors simultaneously attempt to withdraw their money due to concerns about potential bank failures. This collective panic, especially during vulnerable phases of the economic cycle, can result in mass withdrawals, leading to the collapse of numerous banks and possibly triggering a broader financial crisis or instability [13]. The theory thus illustrates a link between DFI and FC by demonstrating how the actions of digitally included depositors, influenced by the economic cycle, can exacerbate financial instability.

### 2.2 The impact of DFI on FC in low FD countries

This section provides a detailed analysis of the channels or mechanisms through which DFI can trigger FC in countries with LFDCs. In the current study, two primary channels through which DFI impacts FC in LFDCs have been identified: (1) restricted access to bank deposits, and (2) limited access to credit and lack of loan diversification [2,5,7].

The issue of low credit accessibility is commonly observed in countries with low FD levels. Therefore, one of the mechanisms through which DFI may trigger FC is restricted access to credit and the lack of loan diversification. This perspective is supported by existing studies such as Khan (2011) [14] and Morgan and Pontines (2014) [9], who analyzed the relationship between financial stability and FI. They measured FI through the ratio of credit allocated to small and medium-sized enterprises (SMEs) and found a direct linear relationship between financial coverage and the stability of the financial system. Siddik and Kabiraj (2018) [15] also examined the relationship between FI and financial stability, using metrics such as the number of SME borrowers relative to the total number of borrowers and the ratio of non-performing SME loans to total loans. They found a positive relationship between FI and financial stability. Oanh et al. (2023) [7] explored the relationship between FI and financial stability using PCA to aggregate individual indicators such as the number of ATMs and bank branches. Their results indicated a negative relationship between FI and financial stability. More recently, Oanh and Dinh (2024) [2] examined the relationship between DFI and financial stability. Using a combination of quantile regression and wavelet analysis, their findings revealed that while DFI has a positive impact on financial stability throughout the study period, this relationship turns negative during global FC.

Another channel through which DFI can lead to FC in countries with low FD systems is the restriction in access to bank deposits. When the number of depositors in the financial system is low, the deposit base of the banking sector becomes small and less diverse. Consequently, banks must rely more heavily on non-core funding sources, which poses significant risks during a crisis [14]. Additionally, a reduced deposit base makes banks more susceptible to sudden withdrawal pressures during times of stress, potentially leaving them without sufficient deposits to absorb economic shocks, thus increasing the risk of a crisis. This viewpoint is supported by existing studies such as Han and Melecky (2013) [8], who examined the relationship between financial stability and FI. They measured FI through access to and usage of bank deposits, finding that higher FI increases the deposit base of banks during crises and contributes to financial system stability. Similarly, Hannig and Jansen (2010) [16] noted that including low-income and marginalized individuals in the formal financial system enhances the deposit base of banks and helps them withstand economic shocks. Dinh (2024) [1] found that increases in the number of ATMs, bank branches, and deposit amounts can enhance FI for women. This not only reduces gender inequality but also promotes economic growth, helping the economy navigate crises. Ozili (2024) [5] analyzed the relationship between FI and FC across 28 countries from 2006 to 2017. Using indicators such as the number of bank depositors, ATMs, and bank branches, the study concluded that FI is one of the factors contributing to FC.

### 2.3 The impact of DFI on FC in high FD countries

This section provides a detailed analysis of the channels or mechanisms through which DFI can trigger FC in countries with high financial development systems. The current study identifies two primary channels through which DFI may contribute to FC in HFDCs: (1) excessive expansion of DFI and (2) excessive debt accumulation.

The first channel through which DFI can trigger FC in countries with high FD systems is excessive expansion of DFI beyond necessary levels. In HFDCs with strong FD, many countries often implement large-scale FI programs by expanding DFI uncontrollably in an attempt to achieve optimal financial inclusion, including extending credit to all segments of the population. For example, the Pradhan Mantri Jan Dhan Yojana (PMJDY) program in India aims to provide formal financial access to everyone, including those with criminal records. This broad expansion of DFI has been linked to financial instability in India, as highlighted by Ozili (2021) [3]. Similarly, in Brazil, widespread DFI programs have been implemented through mobile payments. However, the increased use of technology has also exacerbated social stratification, indirectly increasing the risk of FC. Feghali et al. (2021) [17] examined the relationship between FI, banking market structure, and financial stability. They argue and provide evidence that excessive credit expansion or inclusive credit can undermine financial stability, especially if credit growth occurs recklessly and without consideration of borrowers' ability to repay. In their study, Oanh and Dinh (2024) [2] identified excessive DFI expansion in Vietnam during the global economic crisis as a contributing factor to the FC in the country. This evidence supports the view that uncontrolled expansion of DFI can lead to financial instability and potential crises, especially in countries with existing vulnerabilities in their financial systems.

The final channel through which the level of DFI can cause FC is "excessive debt" [5]. Financial tools such as credit cards are commonly used to drive DFI in highly inclusive financial countries like the United States and the United Kingdom. The easy access to credit cards and other debt instruments in these countries often leads individuals to borrow more than they can repay. When borrowers default and there is no government guarantee for these loans, banks face significant losses, which can lead to the bankruptcy of affected banks. The failure of one bank can trigger the collapse of other banks, leading to a FC.

Based on these arguments, the authors propose the following research hypotheses.

Hypothesis H1 There exists a FD threshold that alters the structure of the relationship between DFI and FC.

Hypothesis H2 The impact of increased DFI reduces FC in LFDCs.

Hypothesis H3 The impact of increased DFI increases FC in HFDCs.

### 2.4 Research gaps

The survey of existing studies on the relationship between DFI and FC reveals several limitations.

Firstly, in terms of research, previous studies have predominantly focused on the relationship between FI and financial stability [18,19]. Recently, Ozili (2024) [5] examined the impact of FI during FC. However, existing literature has not clearly analyzed the relationship between DFI and FC or considered this relationship in the context of FD. This highlights the need for further research to clarify these aspects and gain a deeper understanding of how DFI can affect FC within different FD contexts.

Secondly, in terms of scope, prior studies have covered a range of geographical contexts, such as global studies [5], specific countries like Vietnam [5,7], and regions such as Indonesia and Malaysia (Jan et al., 2023). In contrast, this study uses a dataset of 52 countries worldwide and further divides these countries into different groups based on the threshold variable of financial development (HFDCs and LFDCs). The study aims to explore how the impact of DFI on FC differs between these two groups of countries.

Regarding research methods, previous studies have employed a variety of approaches, including 2SLS, FEM, and AMOS. A common limitation of these methods is their lack of flexibility in adapting to structural changes and their inability to adequately reflect data diversity without appropriate adjustments. The impact of expanding DFI on FC varies based on the level of FC in different countries. In this study, we utilize two combined methods:

PTR: Proposed by Hansen (1999) [11], this model identifies FD thresholds. Based on these thresholds, countries are classified into different groups. This approach allows for detecting structural breaks and varying impacts of DFI on FC according to different levels of FD. Many previous studies have used quantitative methods related to structural breaks with the aim of reassessing the impact of macroeconomic variables on each other. For example, examining the relationship between financial development (FD), renewable energy, and CO2 through the NARDL model [20]; financial inclusion and energy efficiency through the quantile regression model [21]; FD and ecological footprint using threshold cointegration and fractional frequency causality tests [22]; reassessing the impact of coal consumption and non-carbohydrate energy on CO2 emissions using the ARDL method [23]. Additionally, methods such as the Rolling Window-Based Nonparametric Quantile Causality Test [24], ARDL [25], and CS-ARDL [26] also demonstrate advantages in examining nonlinear models. The models above demonstrate advantages when studying variables with causal relationships. In this study, we use PTR, which can clearly show the impact of DFI on FC.

Bayesian Regression Model: This model is advantageous for addressing issues related to small sample sizes, endogeneity, and autocorrelation. It provides a probabilistic framework to examine the relationship between DFI and FC, accommodating data diversity and offering more nuanced insights. By integrating these methods, our study aims to provide a more flexible and comprehensive analysis of how DFI impacts FC across varying FD contexts.

## 3 Research methodology

### 3.1 Data and sample

The countries were selected based on data availability. The research data were collected from three primary sources: the Global Financial Development Index, the Worldwide Governance Indicators from the World Bank (WB), and the Financial Access Survey from the International Monetary Fund (IMF). Missing data were handled using Bayesian linear regression methods as proposed by Van Buuren (2018) [27]. The final sample consists of a balanced panel of 52 countries covering the period from 2004 to 2020. The definitions and measurements of all variables are presented in Table 1.

**Table 1:** Variable description and source.

Symbol	Indicator	Measurement	Source
**Dependent variable**			
Zscore	Financial Crisis	Zscore = (ROA + EA)/(σ(ROA)) where: EA represents equity to asset ratio σROA represents the standard deviation of ROA	WB
**Independent variables**			
DFI	Digital Financial Inclusion	We use the PCA technique to calculate DFI.	Author
1. BRA	The number of bank branches.	Number of commercial bank branches per 100,000 adults.	WB, IMF
2. ATM	The number of ATMs	Number of ATMs per 100,000 adults.	WB, IMF
			WB, IMF
3. OLB	Outstanding loans from commercial banks	The percentage representing the total value of loans provided by commercial banks within a specific nation relative to its GDP.	WB, IMF
4. ODB	Outstanding balance of deposits at commercial banks	The percentage indicating the total value of deposits maintained within commercial banks within a given country in relation to its GDP.	WB, IMF
5. MOBI	Mobile cellular subscriptions	Mobile cellular subscriptions per 100 people	WB
6. INT	Individuals using the Internet	The percentage of individuals in a specific country or region who have access to and utilize the Internet. (%)	WB
7. FBS	Fixed broadband subscriptions	Fixed broadband subscriptions (per 100 people)	WB
**Threshold variable**			
FD	Financial development index	Using 105 indicators from GFDD and 46 from FinStats, experts constructed sub-indices (FID, FIA, FIE, FMD, FMA, FME, FI, FM) and combined them into the overall FD index.	IMF
**Control variables**			
DR	Domestic credit to private sector (% of GDP)	Domestic credit to the private sector represents the share of financial resources supplied to the private sector by depository institutions, excluding central banks, as a percentage of GDP. (%)	WB
INF	Inflation Rate	Annual CPI growth rate (%)	WB
EG	Economic growth	GDP growth per capita (%)	WB
ER	Efficiency Ratio	The proportion of a bank's operating expenses relative to the total of its net interest revenue and other operating income (%)	WB
CAR	Capital adequacy ratio	The capital adequacy of deposit-taking institutions is determined by the ratio of their total regulatory capital to the risk-weighted value of their held assets (%)	WB

### 3.2 Model specification

Based on the bank run theory by Diamond and Dybvig (1983) [13], the relationship between DFI and FC demonstrates how the actions of depositors included in digital platforms, influenced by economic cycles, can exacerbate financial instability. Furthermore, based on previous research by Ozili (2024) [5] and Ozili et al. (2023) [28], the empirical model for examining the impact of DFI on FC is specified as follows:

(1) $${Zscore}_{i,t}=\beta_{o}+\beta_{1}{DFI}_{i,t}\left({FD}_{i,t}<\gamma_{1}\right)+\beta_{2}{DFI}_{i,t}\left({\gamma_{1}<FD}_{i,t}<\gamma_{2}\right)+\beta_{3}{DFI}_{i,t}\left({\gamma_{2}<FD}_{i,t}\right)+\beta_{x}X_{i,t}+{\;\varepsilon }_{i,t}$$

In this model, the dependent variable Zscore represents FC. The variabl DFI denotes digital financial inclusion, which is aggregated using Principal Component Analysis (PCA) and includes 7 components (see Table 1). The vector X consists of control variables, including Domestic Credit (DR), Inflation Rate (INF), Economic Growth (EG), Capital Adequacy Ratio (CAR), and Efficiency Ratio (ER). The authors will further explain the selection of these variables in the empirical model.

### 3.3 Variables justification

Several previous studies have utilized the Z-score of banks to assess FC or the stability of the banking system [[5], [6], [7],[28], [29], [30]]. A high Z-score indicates that the banking system is far from the risk of systemic default or FC, implying that the banking system is stable. This method is distinguished by its ability to clearly identify phases of FC, thereby enhancing accuracy and flexibility in analyzing complex situations. Using the Z-score allows for precise identification of crisis periods, facilitating effective analysis of the impact of independent variables on the likelihood of FC.

$$Zscore=\frac{ROA+EA}{\sigma_{ROA}}$$

where EA represents the equity-to-asset ratio, and σ_ROA represents the standard deviation of ROA. This calculation is performed for each year in countries with at least five bank-level observations.

In previous studies, the measurement of DFI has varied. However, a common finding across these measures is that DFI cannot be captured by a single variable but rather requires a combination of indicators related to the extent of financial access [6]. Therefore, based on earlier research such as Dinh (2024) [1], Oanh and Dinh (2024) [2] and Hai and Dinh (2024) [31], the authors developed a composite measure of DFI using seven components: the number of bank branches (BRA); the number of ATMs; the outstanding balance, which includes outstanding loans from commercial banks (OLB) and the outstanding balance of deposits at commercial banks (ODB); mobile cellular subscriptions (MOBI); fixed broadband subscriptions (FBS); and individuals using the Internet (INT).

$$DFI=W_{1}BRA+W_{2}ATM+W_{3}OLB+W_{4}ODB+W_{5}MOBI+W_{6}FBS+W_{7}\mathrm{INT}$$

Where: DFI is the digital financial inclusion index, with W representing the weight each variable contributes.

**Fig. 1:**
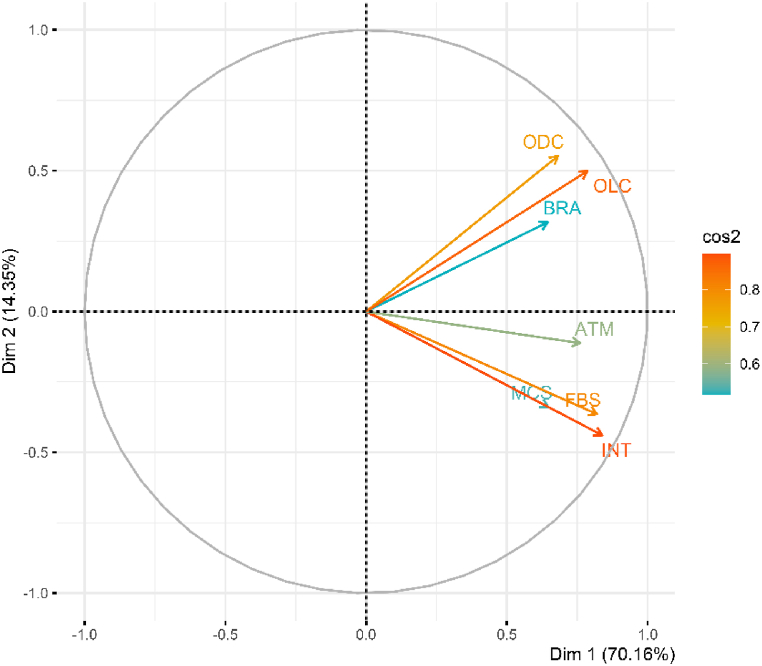
Quality of component variables and eigenvalue.

Source: Author's calculations using R software.

PCA method is deemed suitable for synthesizing the DFI index. PCA is a data transformation technique that reduces a large number of highly correlated variables into a smaller set of uncorrelated variables, with the new variables being linear combinations of the original variables [2]. Appendix 1 presents the correlation results among the variables, indicating a high correlation among the seven component variables, which supports the application of PCA for this study. Additionally, the results in Fig. 1 demonstrate that the quality of the component variables is very high, with the first component explaining 70.16 % of the total variance. Therefore, the results from the seven component variables are satisfactory. Based on the PCA results, the authors calculated the DFI using the equation (∗). It was found that MCS and INT have substantial weights contributing to the formation of the DFI index, with weights of 0.3512 and 0.3430, respectively. The large weight of MCS highlights the significant impact of mobile financial services on the DFI index. Similarly, the weight of INT underscores the importance of technological infrastructure and internet connectivity in promoting digital finance.

DFI=0.6244∗ATM+0.1562∗BRA+0.3530∗ODC+0.4453∗OLC+0.3430∗INT+0.1470∗FBS+0.3512∗MCS(∗)

We also control for macroeconomic factors affecting FC. The Efficiency Ratio (ER) measures banking performance efficiency. Research indicates that banks able to reduce costs relative to income perform better compared to banks with high costs and low income [32]. In the context of a crisis, less efficient banks with higher ER ratios may struggle more to maintain stable operations, thereby increasing the risk of FC. Inflation (INF) can trigger FC through various mechanisms. When inflation rises, it typically leads to price instability, reducing the real value of currency and eroding the purchasing power of consumers and businesses. This can result in decreased consumption and investment, placing pressure on the economy. Additionally, high inflation is often accompanied by higher interest rates, increasing borrowing costs and weakening borrowers' repayment capacity. This may lead to a rise in non-performing loans and defaults, undermining the financial system and potentially pushing banks and financial institutions into distress, ultimately causing FC [7].

A sharp increase in credit to the private sector (DR) may indicate a rapid expansion in bank lending to the private sector. If this lending is not carefully managed, it can lead to a significant amount of high-risk loans, increasing the likelihood of defaults. When these loans are not repaid, it can create substantial financial pressure on banks, potentially leading to FC [33,34].

### 3.4 Research Methodology

PTR model describes structural breaks or jumps in the relationship between variables [11,12]. When the changes in exogenous factors fall within different threshold ranges, independent variables will have varying effects on dependent variables. Applying threshold effects in research not only allows for a more precise description of the impact of independent variables on dependent variables but also examines the influence of exogenous factors on their relationship (Wang et al., 2023). In this study, we emphasize the relationship between DFI and FC at different levels of FD thresholds. Based on the level of the threshold variable, which reflects financial stability, we classify countries into different groups. According to the FD threshold results presented in Table 2, we divide the 52 countries into two groups: 35 LFDCs and the remaining 17 HFDCs. Bayesian regression, known for its strengths in addressing issues related to small samples, endogeneity, and autocorrelation [13], is employed to examine the relationship between DFI and FC across these three groups of countries.

**Table 2:** Overview of descriptive statistics.

Variable	Mean	Std. dev.	Min	Max	Pesaran CD Test	Jarque Bera Test
Zscore	15.9699	7.9675	−0.1872 (Greece)	43.1023 (Panama)	21.190∗∗∗	0.000∗∗∗
DFI	137.8921	63.5803	7.3123 (Rwanda)	296.9971 (Switzerland)	103.857∗∗∗	0.000∗∗∗
FD	0.4567	0.2462	0.0783 (Uganda)	0.9968 (Switzerland)	33.741∗∗∗	0.000∗∗∗
DR	65.5319	41.4984	7.6130 (Uganda)	201.2585 (Denmark)	33.221∗∗∗	0.000∗∗∗
INF	3.8687	4.2550	−4.4475 (Ireland)	51.4609 (Dominican Republic)	57.285∗∗∗	0.000∗∗∗
EG	1.9183	3.6432	−18.8544 (Panama)	23.3047 (Ireland)	88.506∗∗∗	0.000∗∗∗
ER	58.1603	13.2315	18.6540 (Ireland)	237.0542 (Switzerland)	53.131∗∗∗	0.000∗∗∗
CAR	16.4715	3.3603	9.400 (Portugal)	33.800 (Eswatini)	44.440∗∗∗	0.000∗∗∗

In Bayesian statistics, research data is integrated with prior information to generate posterior distributions, allowing results to be interpreted as probability distributions of parameter values, independent of sample size. As a result, Bayesian methods effectively overcome the challenges posed by small sample sizes in studies (Binh et al., 2024). Bayesian and frequentist approaches hold fundamentally different philosophies regarding the treatment of fixed versus random elements, leading to different interpretations of research results. The Bayesian approach treats the observed sample data as fixed, while model parameters are considered random. It estimates the posterior distribution of these parameters by combining the observed data with their prior distribution, and this posterior distribution is then used to interpret results. In contrast, the frequentist approach assumes that the observed samples are random repetitions and treats parameters as fixed but unknown, interpreting results based on the sampling distribution or statistical properties of the data. Essentially, Bayesian analysis provides answers by focusing on the distribution of parameters conditional on the observed data. Beyond addressing small sample sizes, Bayesian regression offers additional benefits in managing issues like endogeneity and autocorrelation [13].

To examine the impact of DFI on FC, Bayesian regression will be conducted through the following three steps:

Prior Assumptions: A normal prior distribution with a mean of zero is assumed for all coefficients, encouraging estimates close to zero. This prior does not bias the results toward positive or negative outcomes.

Likelihood Functions: The likelihood functions for the coefficients are based on normal distributions, ensuring consistency with the model from Equation (1).

Posterior Distribution: We use Markov Chain Monte Carlo (MCMC) methods, particularly the Gibbs Sampler, to estimate the posterior distributions. After generating 15,000 samples, the first 2500 are discarded as burn-in Ref. [6].

## 4 Research findings

### 4.1 Overview of descriptive statistics

**Fig. 2:**
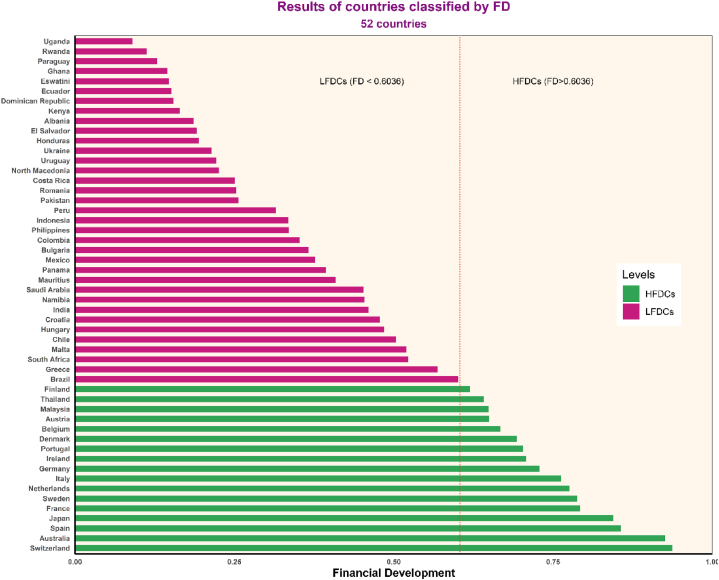
Distribution of 52 countries based on FD levels.

Source: Calculations by the author.

Table 2 presents the descriptive statistics for 52 countries (Fig. 2) over the period from 2004 to 2020. The average Z-score is 15.9699, with Panama having the highest Z-score at 43.1023, indicating the highest level of financial stability and the lowest risk of financial crisis among the sample countries. Conversely, Greece has the lowest Z-score at −0.1872, reflecting the lowest financial stability and the highest risk of financial crisis within the study sample. The average DFI score is 137.8921, with a standard deviation of 63.5803. The lowest DFI score is 7.3123, reported by Rwanda, signifying the lowest level of digital financial inclusion. In contrast, Switzerland has the highest DFI score at 296.9971, indicating the highest level of DFI. The average FD score is 0.4567, with a standard deviation of 0.2462. The lowest FD score is 0.0783, observed in Uganda, reflecting the lowest level of FD. Conversely, Switzerland has the highest FD score at 0.9968, indicating the highest level of FD.

Evaluating cross-sectional dependence (CD) is crucial in panel data analysis. To address this, we employed the Pesaran (2021) [[35], [36]] test, as neglecting cross-sectional dependence can lead to erroneous conclusions. Table 2 presents the results of Pesaran's cross-sectional correlation test, which reveal significant cross-sectional correlations among all variables. the rejection of the null hypothesis in the Jarque-Bera test indicates that the variables do not conform to a normal distribution, which could complicate the use of standard regression models that assume normality. These issues highlight the need for more robust methodologies that can accommodate such complexities in the data. Consequently, we can leverage the advantages of PTR and Bayesian regression to effectively address these challenges, as both methods are well-suited to handle non-normal distributions and capture complex relationships in the data.

### 4.2 PTR results

Initially, we conducted a threshold regression model with three thresholds, testing the null hypothesis: $\beta_{1}=\beta_{2}$ (no threshold effect). We used 300 bootstrap iterations to assess the presence of a threshold effect. If a three-threshold effect was not detected, we proceeded to perform a two-threshold regression, and similarly, a single-threshold regression would be conducted if the two-threshold effect was not present. The results presented in Table 3 indicate that the null hypothesis *H*0 is rejected at the 1 % significance level, suggesting the presence of a single-threshold effect. In other words, the relationship between Z-score and DFI is nonlinear when FD is used as the threshold variable, with the threshold value identified as 0.6036.

**Table 3:** Panel threshold effect test results.

Threshold	Threshold Value	F-value	P-value	10 %	5 %	1 %
**Single**	0.6036	22.05	0.0033	11.3612	13.3759	20.4650

Table 4 presents the regression results on the impact of DFI on Zscore with the threshold value of FD set at 0.6036. The results indicate that when FD ≤ 0.6036, DFI has a positive effect on Zscore with a coefficient of 0.0695, which is statistically significant at the 1 % level. However, when FD exceeds the threshold of 0.6036, the effect of DFI on Zscore becomes negative, with a regression coefficient of −0.0375. This suggests that in LFDCs, DFI can enhance financial stability and reduce the risk of FC. Conversely, in HFDCs, DFI may have adverse effects, increasing the risk of FC. This result supports hypothesis H1, which posits the existence of an FD threshold that disrupts the linear relationship between DFI and Zscore. Compared to the studies by Dinh (2024) [1] and Oanh et al. (2023) [7], where countries were categorized into two groups based on the mean FD (low and high FD groups), this study provides a more nuanced analysis by applying Hansen's (1999) [11] threshold regression method to accurately identify the structural break points. Instead of relying solely on the average FD, this study utilizes structural thresholds to categorize countries, yielding more reliable results. Next, to verify the robustness of the PTR results and address hypotheses H2 and H3, we proceed by dividing the 52 countries into distinct groups based on FD thresholds (see Fig. 2).

**Table 4:** Panel threshold regression results.

Variables	Coefficients	Std	t-value
C	14.0195	2.1978	6.3800∗∗∗
DR	−0.0247	0.0231	−1.0700
INF	0.0571	0.0135	4.2416∗∗∗
ED	0.0671	0.0235	2.8600∗∗∗
ER	−0.0697	0.0144	−4.8500∗∗∗

CAR	0.0643	0.0334	1.93∗∗∗

DFI (FD ≤0.6036)	0.0695	0.0108	6.4124∗∗∗
DFI (FD > 0.6036)	−0.0375	0.0105	−3.5689∗∗∗

The results of the classification of the 52 countries are presented in Fig. 2, which shows a division into 35 LFDCs and 17 HFDCs. Notably, among the 35 HFDCs, Uganda and Rwanda have the lowest FD, while among the 17 HFDCs, Australia and Switzerland have the highest FD, reflecting significant differences in FD levels across countries. With the country groups established, we proceed with Bayesian regression to examine how the relationship between DFI and FC differs across varying FD levels. Bayesian regression addresses issues of endogeneity, autocorrelation, and small sample sizes (in both country groups). Moreover, the results of Bayesian regression are compared with those from the threshold regression analysis. This comparison can provide valuable practical implications and assist in formulating appropriate recommendations.

### 4.3 Bayesian results

The Bayesian regression results demonstrate the impact of DFI on Zscore for the two groups of countries, as illustrated in columns (1) and (2) of Table 5. These results show that the effect of DFI on Zscore is consistent with the direction of impact observed in the PTR analysis (Table 4). The study confirms that in LFDCs, expanding DFI can enhance financial stability and reduce the likelihood of FC. Conversely, in HFDCs, expanding DFI may increase the risk of crises. These findings support hypotheses H2 and H3, indicating that the impact of DFI on FC is contingent upon the level of FD within a country. Compared to Ozili (2024) [5], this study provides further insights into the differences between DFI and FC based on the threshold variable FD. Specifically:

**Table 5:** Bayesian regression results in LFDCs and LFDCs.

**Variables**	**Dependent variable: Zscore**					
	**LFDCs**			**HFDCs**		
	Mean	Std. Dev.	MCSE	Mean	Std. Dev.	MCSE
C	2.1998	0.9402	0.0055	2.4758	0.9519	0.0057
DR	0.0911	0.0212	0.0001	0.0548	0.0148	0.0001
INF	−0.0767	0.0779	0.0005	−0.0897	0.3162	0.0018
ED	0.1315	0.0936	0.0005	0.2754	0.1447	0.0008
ER	0.2082	0.0206	0.0001	0.1112	0.0220	0.0001
DFI	0.0194	0.0097	0.0000	−0.0011	0.0100	0.0001
Acceptance rate	0.9937			0.9993		
Efficiency: min	0.9724			0.9169		
Max Gelman-Rubin Rc	1.0000			1.0000		

For LFDCs, expanding DFI enhances their resilience to FC. Increased access to credit and diversification of loans broaden the deposit base of banks, enabling them to better withstand economic shocks, thereby reducing the risk of bankruptcy or FC. These findings are consistent with the views of Oanh and Dinh (2024) [2], who also argue that expanding DFI contributes to stabilizing the banking system and acts as a shield against macroeconomic shocks. In contrast, for HFDCs, the impact of DFI on FC is positive, with a mean value of 0.0011. This suggests that, when access to credit and financial services is expanded rapidly without adequate risk control measures, it may lead to unsustainable credit growth [7]. While businesses and individuals gain easier access to capital, the lack of careful assessment of repayment capabilities can increase non-performing loans, thereby precipitating FC in these countries. Furthermore, DFI can exacerbate financial crises if the use of bank accounts becomes excessive or redundant. A potential issue arises when individuals and businesses become overly reliant on digital financial services without fully understanding the risks, leading to uncontrolled borrowing or risky investments through digital platforms. This can create bad debt and gaps in the financial system, increasing the risk of a crisis. When digital bank accounts increase rapidly without effective management and supervision measures, it can easily lead to an imbalance in credit supply and demand, directly affecting the stability of the financial system and the economy. This finding aligns with Oanh et al. (2023) [7], who also note that uncontrolled expansion of access to capital in highly developed financial systems diminishes financial stability.

Another advantage of the Bayesian method is its ability to provide the probability of the effect of an independent variable on a dependent variable. This probability indicates the level of confidence regarding the impact of the variables, allowing researchers to not only assess the direction and magnitude of the effect but also draw conclusions based on the probability distribution of the parameters. This offers a more comprehensive view compared to traditional estimation methods [6]. Table 6 presents the probability results of the effect of independent variables on Zscore. In LFDCs, DFI has a positive effect on Zscore with a probability of 97.67 %. Conversely, in HFDCs, DFI has a negative effect on Zscore with a probability of 80.31 %. These high probabilities suggest a significant degree of confidence in evaluating the impact of DFI in both groups of countries during FC.

**Table 6:** Probability of the effect of independent variables on zscore.

**Prob**	**Dependent variable: Zscore**					
	**LFDCs**			**HFDCs**		
	Mean	Std. Dev.	MCSE	Mean	Std. Dev.	MCSE
Prob(Zscore:C) > 0	0.9896	0.1015	0.0005	0.9954	0.0674	0.0004
Prob(Zscore: DR) > 0	1.0000	0.0000	0.0000	0.9999	0.0080	0.0000
Prob(Zscore: INF) < 0	0.8392	0.3675	0.0021	−0.0897	0.3162	0.0018
Prob(Zscore: ED) > 0	0.9200	0.2713	0.0015	0.9711	0.1674	0.0009
Prob(Zscore: ER) > 0	1.0000	0.0000	0.0000	1.0000	0.0000	0.0000
Prob(Zscore: DFI) > 0	0.9767	0.1507	0.0009			
Prob(Zscore: DFI) < 0				0.8031	0.1344	0.0003

Bayesian regression diagnostics indicate that the average acceptance rate for equations involving DFI and FC exceeds 0.5 for all models. The minimum efficiency of the equations in both country groups is above the permissible threshold of 0.01 [[13], [37]]. This suggests that all models meet the required standards of quality and reliability, ensuring the accuracy and dependability of the analysis results. The posterior distributions generated using the Markov Chain Monte Carlo (MCMC) technique must ensure that the samples produced by the MCMC algorithm accurately estimate the target distribution (Binh et al., 2024). Therefore, MCMC diagnostic tools are essential for verifying the convergence of Markov chains and determining when to stop sampling. In this study, the Gelman-Rubin statistic, also known as the Rc coefficient, is used to assess the convergence of Markov chains and the effectiveness index to examine the stopping criterion for MCMC sampling. Table 4, Table 5 show that the R c coefficient for all parameters is less than 1.1. According to Levy (2020) [38], Rc value below 1.1 indicates that the MCMC algorithm has produced representative samples, meaning the Markov chains have converged. Furthermore, unlike traditional statistical methods such as OLS, FEM, REM, which rely on p-values less than 5 % for statistical significance, Bayesian methods use Monte-Carlo Standard Error (MCSE) to assess estimation quality. MCSE measures the deviation between estimates from the Markov chain and the actual target value, rather than solely depending on p-values as in traditional methods. According to Flegal et al. (2008) [39], a lower MCSE value indicates greater stability in the MCMC chain. Values of MCSE less than 6.5 % of the standard deviation are considered acceptable, and those below 5 % are deemed optimal. Based on the analysis results from Table 5, Table 6, all variables meet these criteria. Therefore, it can be affirmed that the Bayesian simulation results in this study are robust and reliable.

## 5 Conclusions and policy implications

This study analyzes the impact of DFI on FC across 52 countries from 2004 to 2020. Two integrated regression methods are employed: PTR and Bayesian Regression. First, PTR method proposed by Hansen (1999) [11] is used to identify structural breaks in the relationship between variables. Bayesian Regression, which addresses issues such as small sample sizes, endogeneity, and autocorrelation, is utilized to investigate whether the effect of DFI on FC differs across countries based on the threshold variable of FD. The PTR results reveal the existence of a threshold in the model. In other words, the relationship between DFI and FC is nonlinear when considering FD as the threshold variable, with the threshold value being 0.6036. Based on this threshold, countries are categorized into two groups: LFDCs and HFDCs. Bayesian Regression results show that the direction of impact is consistent with PTR findings. Specifically, DFI reduces FC in LFDCs, while it increases FC in HFDCs. This suggests that what appears to be beneficial may actually have adverse effects, particularly in the expansion of DFI in HFDCs. Compared to previous studies that only focused on linear relationships, this research provides additional insights into the nonlinear relationship between DFI and FC based on FD levels. Furthermore, one of the contributions of this study is the use of a combination of traditional and probabilistic methods to examine and test the robustness of the results.

From these results, the study proposes the following policy implications for the two groups of countries:

For LFDCs: The positive impact of DFI on Zscore indicates that expanding DFI can reduce the risk of FC. Therefore, governments and financial institutions should enhance efforts to promote investment in digital technology by implementing digital skills training programs, providing capital to businesses and individuals, and facilitating support from financial organizations. These measures will not only improve financial access but also contribute to long-term economic stability. Furthermore, policies should be established to encourage individuals and households to participate in the formal financial sector. For individuals who are resistant, ethical measures such as persuasion can be used. This effort must involve mobilizing resources from provincial and local authorities, where managers have a deeper understanding of each individual in the area.

For HFDCs: In these countries, uncontrolled expansion of DFI could lead to increased FC. Therefore, regulatory agencies must develop stricter regulations and monitoring mechanisms for digital financial activities to mitigate the risk of FC. This includes requiring financial institutions to have clear risk management strategies when adopting digital financial technologies. Additionally, regulatory bodies should conduct regular assessments of the impact of DFI on the banking system, especially during major technology transitions or when there is a rapid increase in digital technology adoption. Furthermore, we recommend that policymakers in these countries reassess their national DFI strategies, focusing efforts on achieving an ‘optimal' level of DFI rather than pursuing a 100 % DFI target, which could destabilize the financial system. Reducing inactive or redundant bank accounts should be a priority for these countries.

This study does not delve deeply into other macroeconomic factors that might influence the research outcomes, such as global monetary policy changes or unforeseen economic shocks. Therefore, future research should broaden the scope of analysis and apply a more diverse range of methodologies to provide a more comprehensive understanding of the impact of DFI.

## CRediT authorship contribution statement

**Huy Nguyen Quoc:** Validation, Supervision. **Dinh Le Quoc:** Writing – review & editing, Writing – original draft, Visualization, Project administration, Methodology, Investigation, Formal analysis, Data curation, Conceptualization. **Hai Nguyen Van:** Validation, Supervision, Software, Resources.

## Data and code availability

Data will be made available on request.

## Funding

The authors acknowledge being supported by the 10.13039/100016831Lac Hong University, Viet Nam.

## Declaration of competing interest

The authors declare that they have no known competing financial interests or personal relationships that could have appeared to influence the work reported in this paper.

## Figures and Tables

**Table:** 

	ATM	BRA	ODC	OLC	FBS	INT	MCS
ATM	1						
BRA	0.5149	1					
ODC	0.3309	0.3776	1				
OLC	0.4971	0.5323	0.7857	1			
FBS	0.5934	0.4254	0.3748	0.4437	1		
INT	0.5941	0.3319	0.3796	0.4660	0.868	1	
MCS	0.3785	0.2400	0.3306	0.3737	0.4529	0.6221	1
